# Estimating the incidence of adverse events in Portuguese hospitals: a contribution to improving quality and patient safety

**DOI:** 10.1186/1472-6963-14-311

**Published:** 2014-07-18

**Authors:** Paulo Sousa, António Sousa Uva, Florentino Serranheira, Carla Nunes, Ema S Leite

**Affiliations:** 1 National School of Public Health, Universidade Nova de Lisboa, Avenida Padre Cruz, 1600-540 Lisboa, Portugal; 2 CMDT – Centro de Investigação em Malária e Doenças Tropicais – Saúde Pública, Lisboa, Portugal; 3 Centro Hospitalar de Lisboa Central, Lisboa, Portugal

**Keywords:** Patient safety, Medical errors, Hospitals, Quality of care, Adverse events

## Abstract

**Background:**

Several review studies have shown that 3.4% to 16.6% of patients in acute care hospitals experience one or more adverse events. Adverse events (AEs) in hospitals constitute a significant problem with serious consequences and a challenge for public health. The occurrence of AEs in Portuguese hospitals has not yet been systematically studied. The main purpose of this study is to estimate the incidence, impact and preventability of adverse events in Portuguese hospitals. It is also our aim to examine the feasibility of applying to Portuguese acute hospitals the methodology of detecting AEs through record review, previously used in other countries.

**Methods:**

This work is based on a retrospective cohort study and was carried out at three acute care hospitals in the Administrative Region of Lisbon. The identification of AEs and their impact was done using a two-stage structured retrospective medical records review based on the use of 18 screening criteria. A random sample of 1,669 medical records (representative of 47,783 hospital admissions) for the year 2009 was analyzed.

**Results:**

The main results found in this study were an incidence rate of 11.1% AEs, of which around 53.2% were considered preventable. The majority of AEs were associated with surgical procedures (27%), drug errors (18.3%) and hospital acquired infections (12.2%). Most AEs (61%) resulted in minimal or no physical impairment or disability, and 10.8% were associated with death. In 58.6% of the AEs’ cases, the length of stay was prolonged on average 10.7 days. Additional direct costs amounted to €470,380.00.

**Conclusion:**

The magnitude of these results was critical, reinforcing the need of more detailed studies in this area. The knowledge of the incidence and nature of AEs that occur in hospitals should be seen as a first step towards the improvement of quality and safety in health care.

## Background

Adverse events (AEs) occur with alarming frequency in healthcare. These events represent significant losses from a clinical, economic and social perspective [1-3]. To learn from these events and improve safety, they must be identified, measured and their causes found. Healthcare providers and researchers are searching for an accurate, reliable and low cost method to identify and measure AEs in hospital and other settings. The method developed in the Harvard Medical Practice Study, in the 90s, is the one most often used for national AE studies [4].

Many countries, like Portugal, have taken initiatives over the past decade to address safety problems in health care [3,5,7,8]. The US report “To Err is Human” marked an acceleration in programmes and actions for increased patient safety initiated by health care policy makers, health care professionals and managers [9]. As far as we know there have been no studies to date on the occurrence, nature, preventability and impact of AEs in Portuguese hospitals.

Studies based on the “Harvard method” have been carried out in several countries with different results, finding evidence of 3.4% to 16.6% of patients in acute care hospitals experiencing one or more AEs [10-17]. The results of these studies give data on a critical aspect of hospital performance and stimulate patient safety improvement of each country.

The aims of this study were to estimate the incidence, nature, preventability, cost and impact of adverse events in Portuguese hospitals. This was an exploratory study that also intended to examine the feasibility of applying to Portuguese acute hospitals the methodology previously used in other countries.

## Methods

This work followed a retrospective cohort study design. The methods were based on the protocol used in the Harvard Medical Practice Study [4,11,18] with modifications introduced in subsequent studies undertaken in the United Kingdom, New Zealand, Canada, the Netherlands, Sweden, Brazil, and, more recently, in a sample of 26 hospitals from eight developing and transitional countries [10-12,15-18].

The study was carried out in three public hospitals of the Lisbon Administrative Region (these hospitals are acute care hospitals; with 785 beds, 450 beds and 220 beds respectively; an emergency department, intensive care units and a high surgery volume). Although the participating hospitals were selected by convenience, they reflect the major characteristics of other public hospitals in Portugal regarding dimension (number of beds), emergency department 24 hours per day, intensive care units, medical and surgical departments and casemix index of patients treated. No specialty hospitals (e.g. Pediatric, Oncology, Obstetric) were included in the study. A global random sample of 1,669 medical records was used, representative of 47,783 (3.5%) hospital admissions, between 01 January 2009 and 31 December 2009, fulfilling the inclusion criteria for this study. At each hospital a simple random sample was selected assuming the number of hospital admissions, an incidence of AEs of 8% (based on The Canadian Adverse Events Study) and a confidence level of 95% [12]. The sampling frame included all admissions of patients over 18 years old who had a minimum stay in hospital of 24 hours. Hospital admissions with a primary diagnosis related to psychiatry were excluded. Oversampling was carried out with the expectation that 10% of medical records would be unusable. A two-stage structured retrospective medical records review was carried out based on the use of 18 screening criteria (Table 1).

**Table 1:** Screening criteria and number of medical records showing evidence of one category of adverse event

**Criteria**	**n**	**%**
Hospital-incurred patient injury	101	22.4
Unplanned readmission after discharge from index admission (12 months)	81	18.0
Unplanned admission related to previous healthcare management	43	9.5
Hospital-acquired infection or sepsis	40	8.7
Adverse drug reaction	31	6.9
Unplanned return to the operating room	29	6.4
Any other undesirable outcomes not covered in this list of criteria	28	6.2
Unexpected death	20	4.4
Unplanned transfer from general care to intensive care	17	3.8
Other patient complications	16	3.5
Cardiac or respiratory arrest	14	3.2
Unplanned removal, injury or repair of an organ during surgery	10	2.2
Unplanned transfer to another acute care hospital	6	1.3
Development of neurological deficit not present on admission	6	1.3
Inappropriate discharge to home	6	1.3
Dissatisfaction or correspondence indicating litigation	2	0.44
Injury related to abortion or delivery	1	0.22
Dissatisfaction with care documented in the patient’s medical record	0	0.0
Total	451	100%

The criteria are not mutually exclusive; i.e. one patient can have more than one criterion (in 365 positive medical records there were 451 criteria).

In the first stage, a group of six nurses (two from each hospital, with a minimum of five years experience in clinical audits) assessed the medical records, looking for the presence of, at least, one of the 18 criteria for the presence of a potential adverse event. In stage two, a group of five physicians (one Cardiologist, one Neurologist, two Surgeons, one Internal Medicine, with a minimum of five years experience in clinical codes and in clinical audits) reviewed each positive record in order to confirm the presence of an adverse event, to estimate its impact and determine its preventability, according to the definition established previously. The degree of agreement between the reviewers in each stage was calculated (assessed on a random sample of 10% of medical records) by using kappa coefficient. Neither nurses nor doctors knew the previous classification of their colleagues.

The timing of the AEs in relation to the hospital admission is an important methodological issue. We considered AEs that occurred during the index hospital admission and that were detected during either the index or subsequent hospital admissions over the following 12-month period (in the same hospitals).

Similarly to other studies, physicians estimated, based on evidence in the medical record and their professional judgment, the impact of AEs in two different ways: i) the degree of physical impairment or disability at discharge (minimal, moderate or permanent), or death; ii) the number of additional hospital days directly attributable to AEs.

The costs related to the additional length of stay were estimated based on official accounting data from all the NHS hospitals, providing information on daily costs. This value includes hospital daily costs, namely physicians' and nursing staff, lab tests and exams, medication, housing and overhead [19].

Using professional judgment and based on the information of medical records, the physician reviewers also classified the preventability of each AE using a six-point scale (1- virtually no evidence of preventability; 2- slight to modest evidence of preventability; 3- Preventability not quiet likely less than 50/50, but “close to call”; 4-preventability more than likely 50/50; 5- strong evidence of preventability; and 6 -virtually certain evidence of preventability. The preventability of an adverse event was considered with a score > = 4).

SPSS (version 19) was used for data processing and for statistical analysis. The study was approved by the Ethics Committee of participating hospitals.

## Results

**Figure 1:**
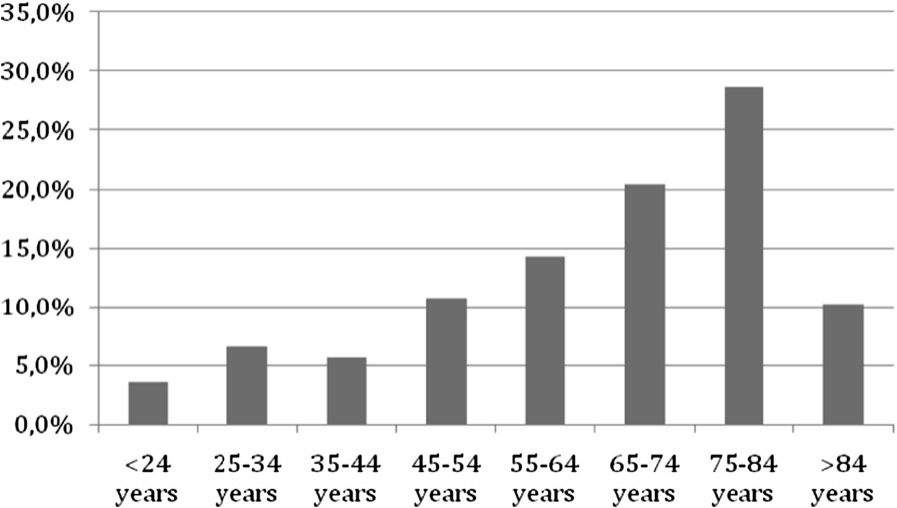
Percentage distribution of adverse events by age group.

One or more of the criteria for an adverse event were identified in 365 out of the 1.669 medical records reviewed (22%). Of these 365 records, those which passed to the second screening, 186 (51%), were confirmed as an AE, an overall incidence rate of 11.1% (186/1669) with a 95% CI (9.6%; 12.6%). The highest proportions of AEs identified were related to surgical procedures (27.0%), drug errors (18.3%) and hospital-acquired infection (12.2%). Most of the AEs (59.2%) occurred in patients aged 65 or over (Figure 1).

Most (61%) of the AEs resulted in no physical or minimal impairment or disability, and were satisfactorily resolved during the admission, or within one month from discharge. Nevertheless, the criteria applied estimated that 5.4% of the AEs resulted in permanent disability according to the definition and 10.8% associated with death (Table 2).

**Table 2:** Degree of physical impairment or disability at discharge

	**n**	**%**
Minimal impairment or disability, recovery within 1 month	113	61.0%
Moderate impairment or disability, recovery within 1-12 months	8	4.1%
Permanent impairment or disability	11	5.7%
Death	20	10.8%
Unable to determine	34	18.4%
Total (of AEs)	186	100%

We found that most of the patients, 109/186 (58.6%), who experienced AEs incurred extra bed days in hospital (a total of 1,166 extra days, an average of 10.7 days per patient, ranging from 1-70 days), with additional total costs of €470,380.00 for all three hospitals together.

Concerning preventability, 53.2% were classified as preventable (Table 3).

**Table 3:** Level of preventability of the AEs

	**n**	**%**	**Preventability**
Virtually no evidence of preventability	43	23.1%	46.8% not preventable
Slight to modest evidence of preventability	23	12.4%	
Preventability not quite likely (less than 50/50, but “close call”)	21	11.3%	
Preventability more than likely (more than 50/50 but “close call”	26	14.0%	53.2% Preventable
Strong evidence of preventability	51	27.4%	
Virtually certain evidence of preventability	22	11.8%	
Total	186	100%	100%

The reliability of the assessment of the screening criteria performed by nurses (first screening) was considered good – substantial agreement (k = 0.63; IC 0.43; 0.79 and p < 0.001; 83.5% agreement). Among doctors (second screening), the reliability of determination of AEs was also good (k = 0.78; IC 0.49; 1 and p < 0.001; 86% agreement) and that of preventability was considered fair (k = 0.58; IC 0.23; 0.94 and p = 0.009; 79% agreement) (Table 4).

**Table 4:** Reliability of assessment between reviewers in the first and the second screening

**Question**	**Screening**	**K statistic**	**CI 95%**	**p-value**
Presence of one or more positive criteria in the medical record analyzed	Screening 1	0.63 (83.5% agreement)	(0.43; 0.79)	< 0.001
AE confirmed by physician	Screening 2	0.78 (89% agreement)	(0.49; 1)	< 0.001
Level of preventability (scale 1–6)	Screening 2	0.58 (79% agreement)	(0.23; 0.94)	0.009

## Discussion

We used retrospective medical record review following the HMPS methodology in order to assess the nature, incidence, and clinical and/or economic impact of adverse events and to provide some information on their causes. Studies in many countries have followed the same methodology and have come to broadly similar conclusions [10-12,14-17]. Rates of AEs in most recent studies lie between 8% and 12%, a range now accepted as being common in the healthcare systems of developed countries.

In this study, we found an incidence of AEs of 11.1% of which 53.2% were considered as preventable. The assessment of preventability is one challenge for these studies, and indicates the potential gains to be achieved by improvements. For this reason, analysis of each case is needed to decide prevention strategies. On the other hand, the classification of preventable AEs, while using a clear criterion and standard, still involves a subjective element and may vary with the expertise, practical experience of the physician and the way the data is registered in the medical record. Some authors argue that knowing the outcome and its severity may influence the judgment of causation and preventability and that the bias element is likely to be in the direction of overestimation of the rate of “preventable” AEs (hindsight bias) [20-22]. Nevertheless, our results are similar to the findings of previous studies, particularly those of the UK (incidence10.8% and 52% preventable), New Zealand (incidence 11.3% and 61.6% preventable) and the Danish study (incidence 9.0% and 40.4% preventable) [10,11,23].

One finding was that 59.2% of patients who experienced an AE were 65 years old or older. Relatively little attention has been paid to patient safety in older people although they are particularly vulnerable to healthcare error and harm [24,25]. Moreover, older people are more likely to suffer from multiple health conditions, receive multiple treatments, and stay longer in hospital. A longer stay increases the risk of all complications of hospitalization. In the last decades more people are living longer and this trend is likely to continue. For all these reasons, it is our opinion that safety for older patients should receive higher priority in research and in safety prevention strategies.

The majority of AEs (61%) were not documented as causing serious consequences for the patient. They did not result in any significant physical impairment or disability, and were resolved during the admission or, within one month from discharge. This is a similar percentage to that reported in other studies [3,10,12,13,17]. However, a meaningful proportion of patients died (10.8%) or experienced a permanent disability as a result of their AEs (5.4%).

The economical impact of adverse events has been gaining particular attention in the last years [26,27]. The report of the Institute of Medicine estimates that, in the United States, the total national costs associated with adverse events represent approximately 4% to 6% of national health expenditures [9]. In Britain, the cost of preventable AEs, in lost bed days alone, was estimated in one billion (Pounds) per year [24]. In the Netherlands (the Dutch adverse events study) the authors concluded that 3% of all bed days and 1% of the total health budget could be attributed to preventable AEs (costs of direct hospital care, mainly additional time in hospital) [20]. In our study, most of the patients (58.6%) who experienced AEs prolonged the length of stay in hospital on average 10.7 days, with additional direct costs of €470,380.00. Extrapolating this estimated value to the population of the study (all admission of the three hospitals in 2009) the costs varied between €1,290.310 and €1,691.643. The overall real costs are higher, as these estimates do not include additional treatments in ambulatory care or other hospitalizations (which are related to the same AE), costs to patients (e.g. medication in ambulatory) or any societal costs (e.g. absence from work, premature death). The costs of prevention, however, may be significant, especially if organizations or their health systems do not have effective implementation capacity [27].

In addition to the costs of unnecessary suffering caused to patients and families, the costs and savings of adverse events are a critical issue in Portugal, which will be experiencing a challenging economic situation for at least the next five years [28] . As with some other European countries, the economic rationale is increasing for selecting effective strategies and implementing them in a context which rewards safety improvement [27]. Including an economic dimension to the knowledge gained from research into quality and safety is an important part of the health system reform that is being implemented in Portugal [5,12,29,30]. Moreover, it could be a starting point for specific interventions in the improvement of patient safety and it may help to prioritise research areas for the near future [6,20,27,31].

There are limitations to the study. These include those applying to retrospective studies, such as information bias and hindsight bias [15,21,22]. We observed that the quality of patient records could be better, although this did not significantly limit the study. Retrospective patient record studies still represent the “gold-standard” method for assessing incidence and monitoring the frequency of AEs [13,20,31,32] and the use of similar methods to those used elsewhere allows comparisons to be made. However, for a more comprehensive assessment of safety, other methods also need to be used, including reporting and learning systems, root-cause analyses, failure mode effects analyzes and morbidity and mortality reviews [13,33].

The reliability of the review process is another critical element in these studies [34,35]. In our study, the level of agreement (Kappa statistic *– k value*) between nurses was considered good - substantial agreement (k value 0.63) and is related to the identification of the presence of at least one positive criterion in the first screening. This value is in line with those found in other studies [11-13,15]. Among doctors (in the second screening), the reliability of determination of AEs was also considered good - substantial agreement with k value of 0.78 and fair concerning their preventability, with k value of 0.58. For the second screening, the k value is slightly higher than those found in other studies undertaken at national level (e.g. Canada, New Zealand, Sweden and The Netherlands). These results must be carefully interpreted, due to the small number of reviewers, cases and hospitals involved in this study [36,37].

## Conclusions

In Portugal, there is an overall awareness and a growing concern about patient safety issues. This study suggests that AEs in three Portuguese hospitals affect nearly one in ten patients and results in considerable avoidable suffering and economic costs. With local evidence of the size of the problem, staff is more motivated to act, especially if effective interventions to reduce adverse events can be selected and implemented to target those AEs that are prioritized, namely those resulting from surgical procedures, drug errors and health-acquired infections.

If interventions can be implemented and demonstrated to be cost-effective, then more healthcare managers and policy makers may begin to view improvements in safety as an investment rather than an expense. This can also speed changes in incentives so as to reward safety and quality. Local evidence of the size and nature of the problem and its clinical and cost impact is one of the first steps toward this desirable change.

## Competing interests

The authors declare that they have no competing interests.

## Authors' contributions

PS coordinated the study (with ASU), was involved in the design of the study, carried out the data collection, analyses and interpretation of the results and drafted the manuscript. ASU coordinated the study (with PS), was involved in the design of the study and helped to carry out the data collection, analyses and interpretation of the results and also drafted the manuscript. FS was involved in the design of the study advised on the analyses and critically revised the manuscript. CN carried out the statistical analyses and reviewed the parts of the manuscript that involved the statistical analyses. ESL was involved in the design of the study and critically revised the manuscript. All authors were involved in revising and approving the final manuscript and accept responsibility for the data presented.

## Pre-publication history

The pre-publication history for this paper can be accessed here:

http://www.biomedcentral.com/1472-6963/14/311/prepub
